# Slipped Upper Femoral Epiphysis: Are We Missing the Point?

**DOI:** 10.7759/cureus.3394

**Published:** 2018-10-01

**Authors:** MN Baig, Orna A Glynn, C Egan

**Affiliations:** 1 Trauma & Orthopaedic Surgery, Galway University Hospital, Galway, IRL

**Keywords:** sufe, hip pain

## Abstract

Slipped upper femoral epiphysis (SUFE) is one of the most common orthopaedic conditions in adolescents. SUFE typically presents as hip pain and limping, but it may present deceptively as knee pain or contralateral hip pain. We discuss a case of a child with a deceptive presentation of SUFE resulting in delayed diagnosis.

## Introduction

Slipped upper femoral epiphysis (SUFE) is characterized by the slippage of the proximal femoral metaphysis anteriorly and superiorly relative to the epiphysis. SUFE is also known as a slipped capital femoral epiphysis. The average age of its manifestation is 13.4 years in boys and 12.2 years in girls [1]. It has a higher incidence in boys, especially in obese children. We describe a case of a 12-year-old boy who presented with the late presentation of an unstable SUFE.

## Case presentation

**Figure 1:**
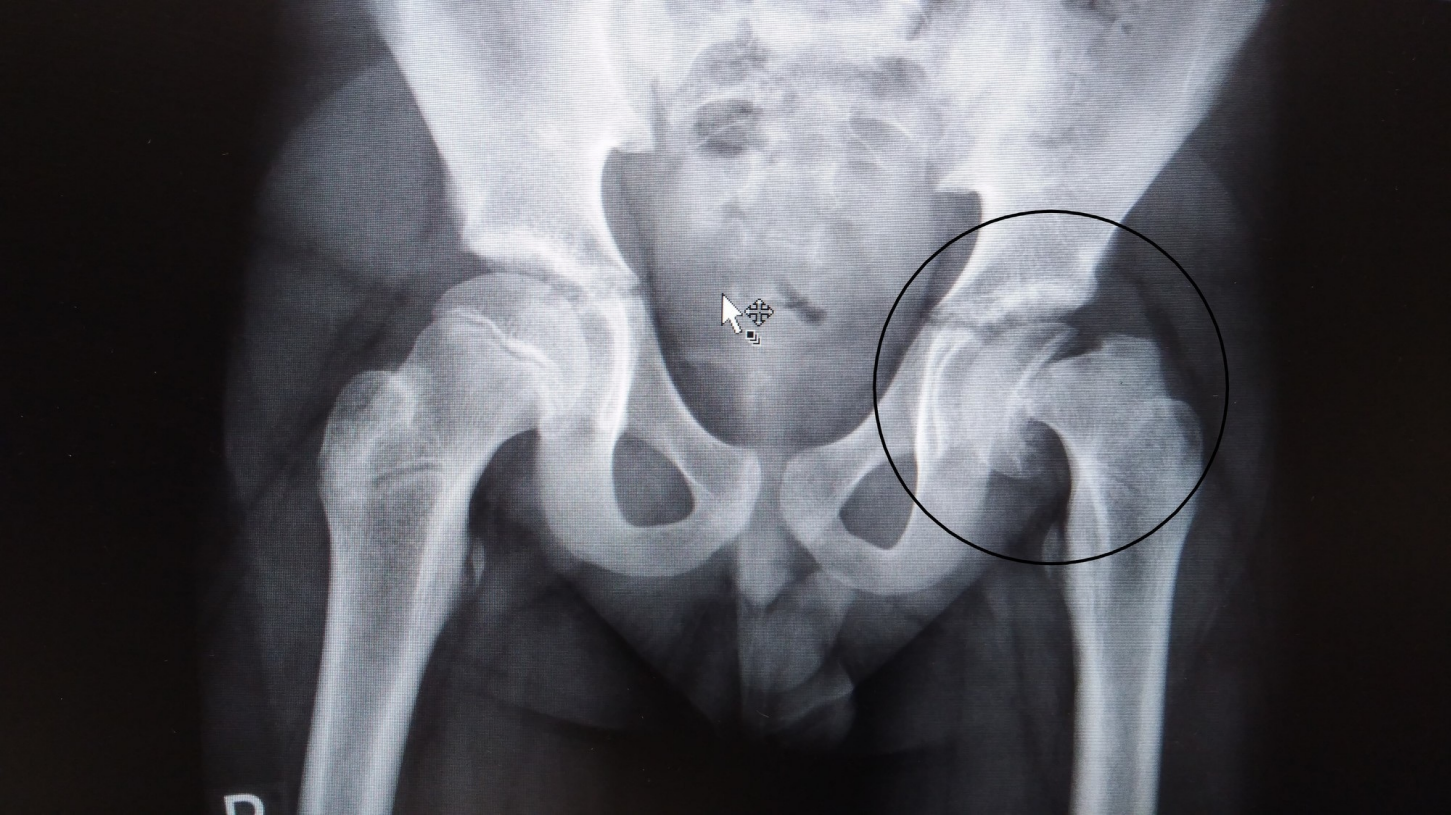
X-ray left hip with slipped epiphysis. X-ray showing complete slippage of the epiphysis.

A 12-year-old boy was referred to our hospital from a peripheral hospital with a diagnosis of SUFE of the left hip. He initially presented to the peripheral hospital with left knee pain and limping two weeks prior. He went to the local hospital where the accident and emergency department personnel obtained knee X-rays showing no abnormality. He was given analgesia, advised bed rest, and was treated as having a soft tissue injury. The knee pain and limping did not resolve. The child presented to the local hospital again after four days with the same concern, and they obtained new knee X-rays. They also performed a clinical examination of the knee and found no abnormality. The analgesia was changed, and they advised further bed rest. The child’s symptoms persisted. He presented again with the knee pain, and the orthopaedic team was asked to review him. The orthopaedic team ordered bilateral knee X-rays and a pelvic X-ray along with a computed tomography scan of his pelvis. The scans confirmed the left hip slipped epiphysis (Figure 1).

**Figure 2:**
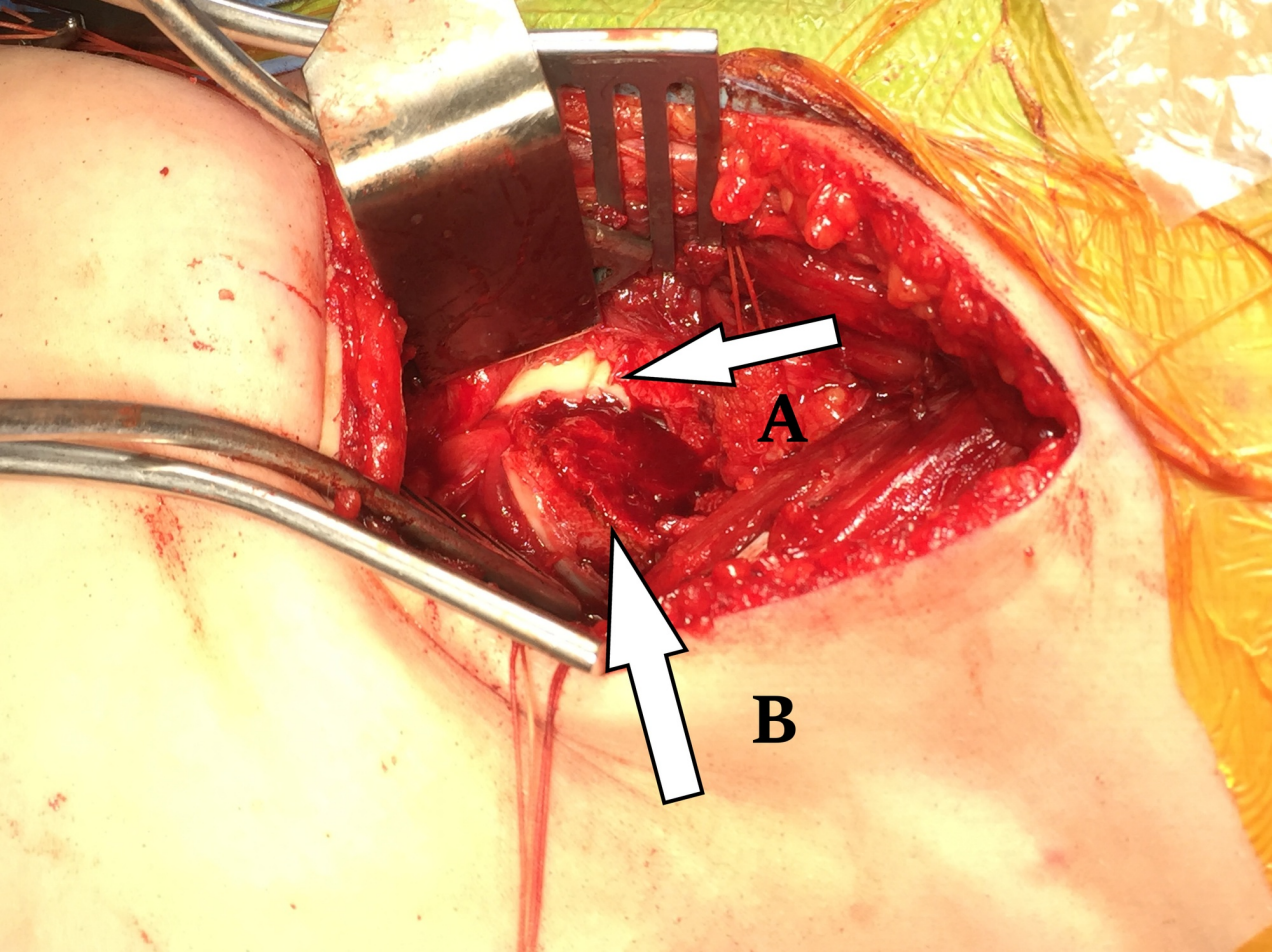
Open reduction of slipped epiphysis. The picture shows intra-operative view of the hip. A- Epiphysis B- Metaphysis

**Figure 3:**
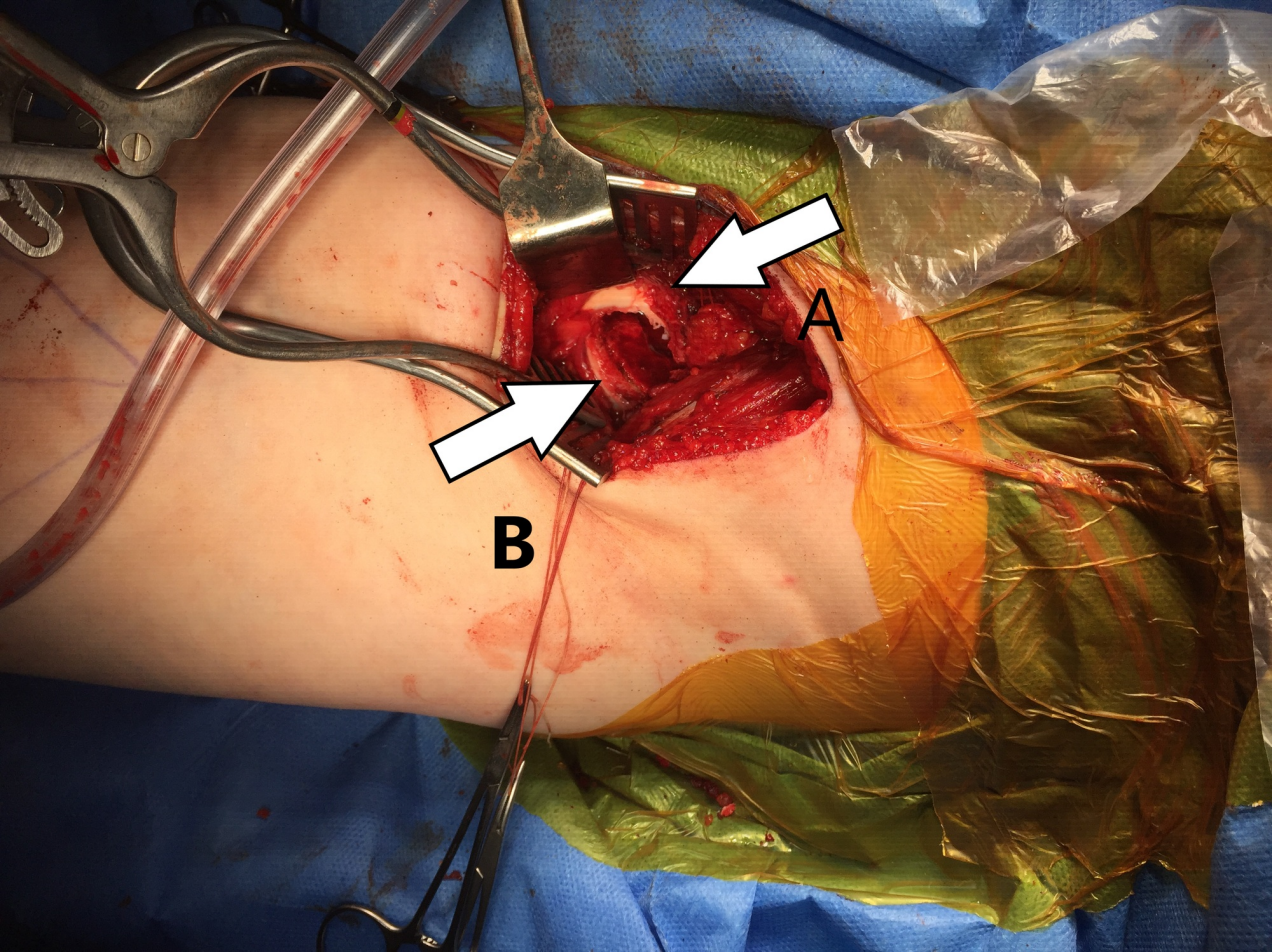
Left hip intra-operative. Slippage visible in the picture. A- Epiphysis B- Slipped metaphysis

**Figure 4:**
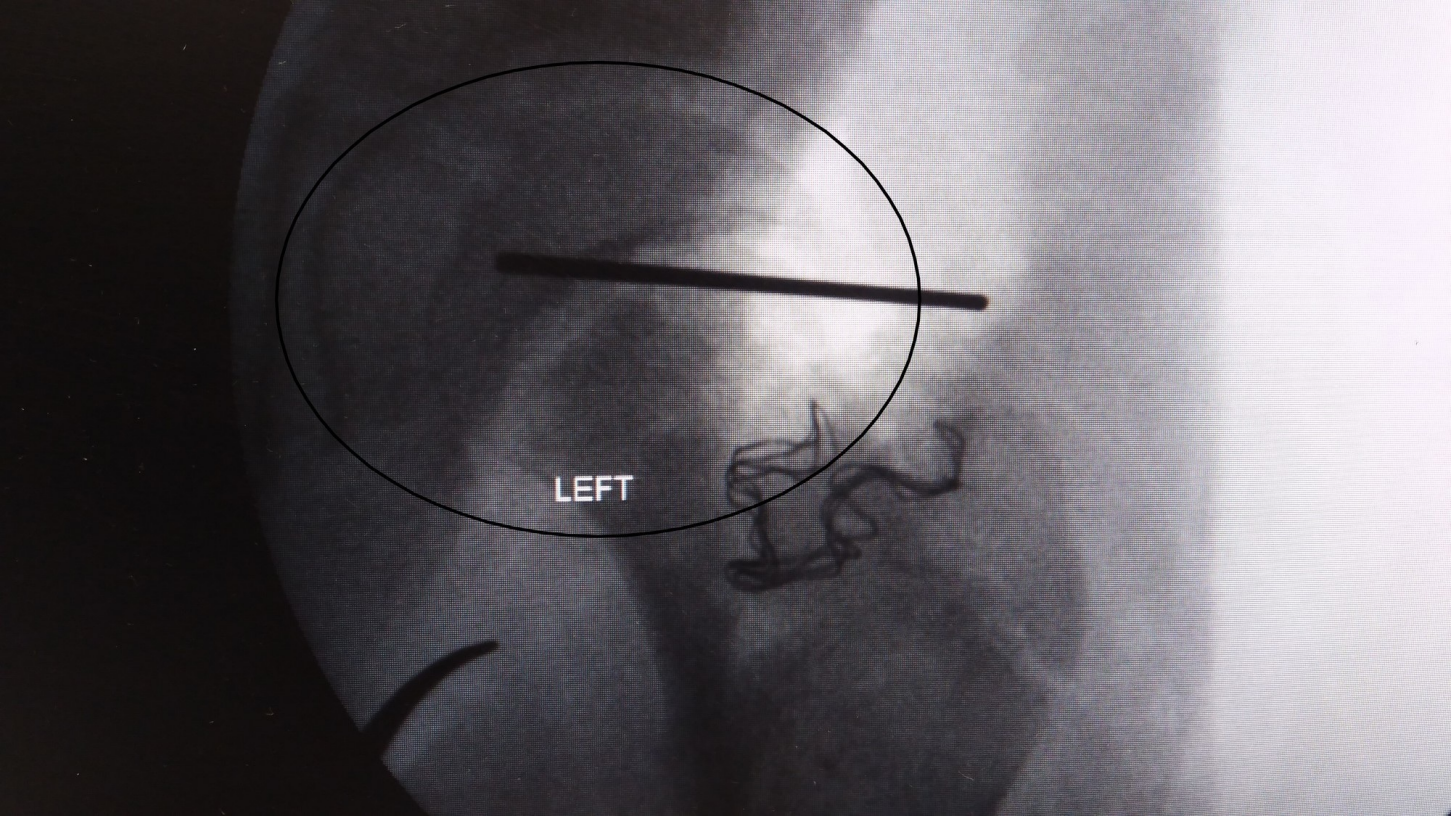
X-ray left hip intra-operative. X-ray left hip showing reduced with Kirschner wire.

**Figure 5:**
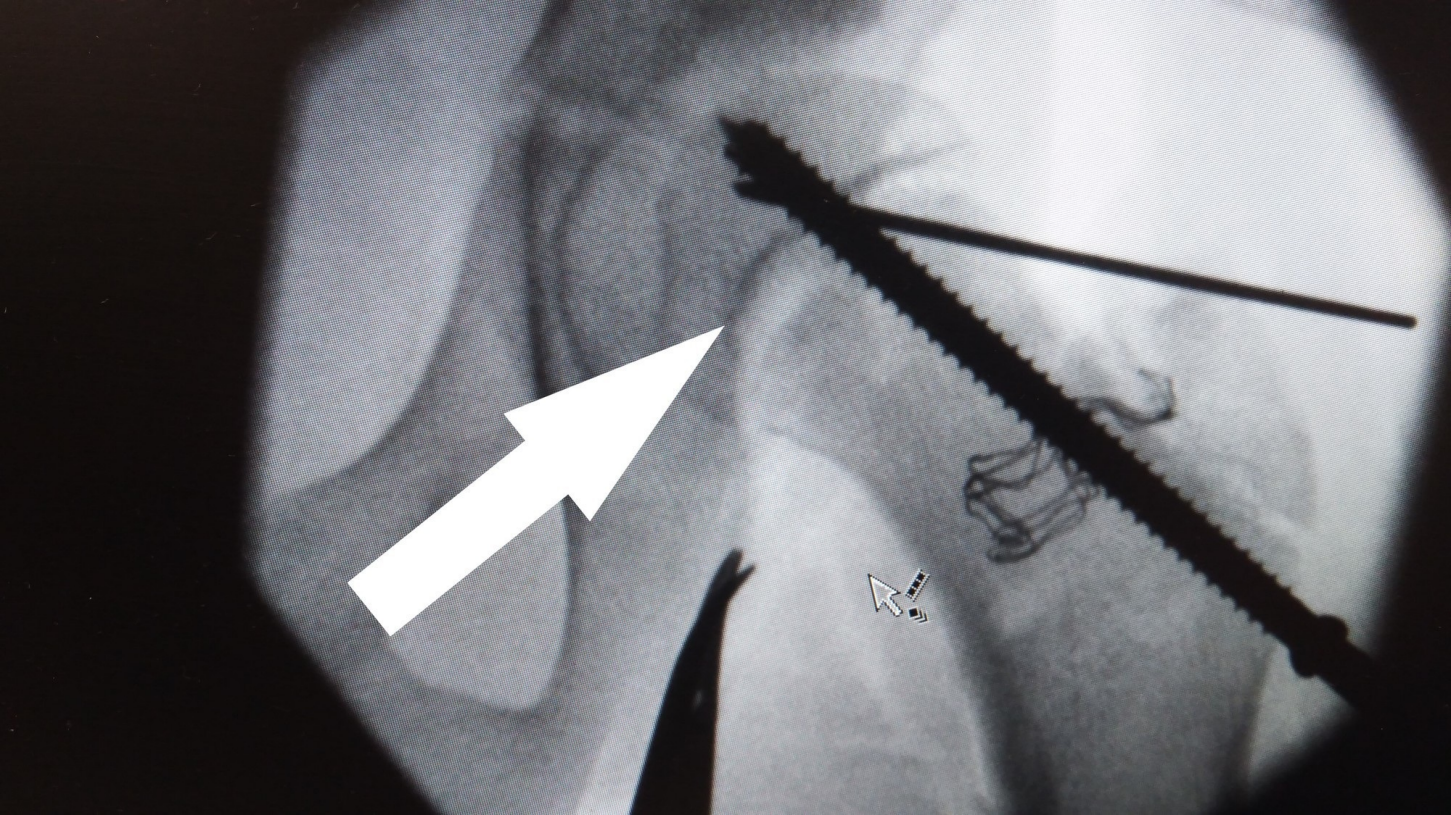
X-ray left hip intra-operative. Final X-ray intra-operative.

**Figure 6:**
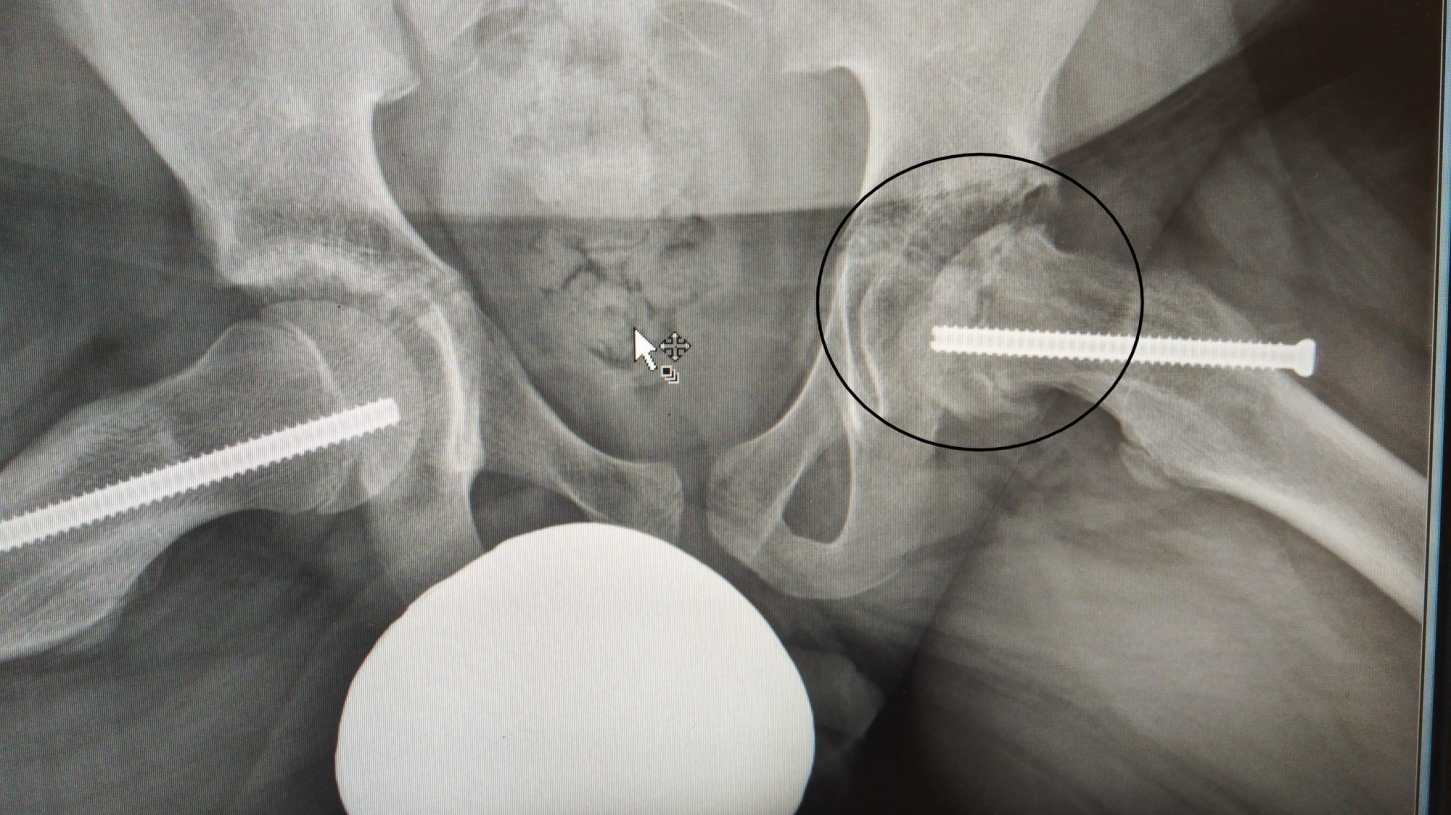
Frog lateral X-ray. Frog lateral X-ray pelvis, the encircled left hip showing avascular necrosis.

He was transferred to our hospital as we had the paediatric orthopaedics resources. The scans confirmed the Loder Classification of unstable and a severe Southwick Slip Angle Classification, with more than 50% slippage. He was admitted and taken to the operating theatre the next day. The intra-operative pictures confirmed almost 100% slippage of the metaphysis (Figures 2, 3). He underwent the open epiphyseal reduction and fixation using the modified Dunne procedure (Figures 4, 5). He was monitored via follow-up in the clinics after he was discharged. Approximately three months after the procedure, he developed signs of avascular necrosis (Figure 6).

## Discussion

Obesity is considered the major predisposing factor for SUFE development, and other factors include acetabular retroversion and femoral retroversion [2]. These conditions cause abnormal mechanical forces on the physis. Every physis has four zones; the epiphysis slippage in SUFE occurs in the hypertrophic zone. The SUFE name is misleading, as the name indicates there is slippage of the epiphysis, but actually the epiphysis maintains its normal position and the metaphysis moves anteriorly and externally rotates [3].

While different classification systems exist, the most common classifications are the Loder Classification (Table 1) and the Southwick Classification. If the diagnosis of SUFE is delayed or missed, the condition can cause complications like osteonecrosis of the femoral head, chondrolysis, residual proximal femoral deformity, leg length discrepancy, degenerative arthritis and femoral-acetabular impingement in later life [4,5]. If the diagnosis is delayed, as seen in this case, because of atypical presentation, the condition can proceed to unstable slippage and avascular necrosis even after surgical intervention and correction.

**Table 1:** Lodder classification. Lodder classification according to slippage of femoral epiphysis. AVN: Avascular necrosis.

Stable	Less severe slippage, able to weight bear, minimal risk of AVN < 10%, good prognosis.
Unstable	More severe slippage, unable to weight bear, high risk of AVN 24%–47%.

## Conclusions

Whenever a limping adolescent child presents, the initial presenting concerns such as knee pain can be deceiving. Therefore, physicians should be vigilant and rule out all the provisional diagnoses. It is important to diagnose SUFE before it becomes unstable as there is a high chance the condition will proceed to avascular necrosis.
